# Osteoma Cutis of the Face in CBCT Images

**DOI:** 10.1155/2017/8468965

**Published:** 2017-05-30

**Authors:** Daniah Alhazmi, Fatma Badr, Fatima Jadu, Ahmed M. Jan, Zainab Abdulsalam

**Affiliations:** ^1^Oral Diagnostic Sciences Department, Division of Oral and Maxillofacial Radiology, Faculty of Dentistry, King Abdulaziz University, P.O. Box 21589, Jeddah, Saudi Arabia; ^2^Oral and Maxillofacial Surgery Department, Faculty of Dentistry, King Abdulaziz University, Jeddah, Saudi Arabia

## Abstract

Osteoma cutis (OC) is a rare benign disorder where osseous nodules form in the reticular layer of normal skin. These nodules are formed by the deposition of lamellar bone and are characterized by osteocytes in the core and osteoclasts around the periphery. Interpretation of osteoma cutis cases has always been challenging especially using conventional two-dimensional (2D) radiographs, owing to difficulty in localization. Cone beam CT (CBCT), with its three-dimensional (3D) capabilities, offers a great tool to help detect and diagnose these calcific entities. We report a case of miliary type OC incidentally detected in the maxillofacial region using CBCT imaging.

## 1. Introduction

Osteoma cutis (OC) is a rare benign disorder where osseous nodules form in the reticular layer of normal skin [1]. These nodules are formed by the deposition of lamellar bone and are characterized by osteocytes in the core and osteoclasts around the periphery [2]. The etiology of this condition remains unclear [3]. Females are more commonly affected especially during the second and third decades of their lives [3]. The disease is classified into primary and secondary forms. Primary osteoma cutis (POC) is not associated with any predisposing factors or conditions, whereas secondary osteoma cutis (SOC) is a consequence of metabolic alterations that increase blood calcium levels [4–7].

Cone beam computed tomography (CBCT) is a three-dimensional (3D) imaging modality that is widely used by dentists for a variety of indications [8]. It offers many advantages such as elimination of superimpositions and submillimeter spatial resolution [8]. More importantly, the radiation dose from certain CBCT examinations using specific exposure factors can be comparable to a single panoramic examination. Furthermore, the radiation dose from specific CBCT examinations can be as low as one-sixth of that of conventional multidetector CT [9].

Interpretation of osteoma cutis cases has always been challenging especially using conventional 2D radiographs, owing to difficulty in localization. Cone beam CT, with its 3D capabilities, offers a great tool to help detect and diagnose these calcific entities. Safi et al. in a recent publication reported the prevalence of osteoma cutis as an incidental finding detected on CBCT to be 2.27% [10]. They also classified the imaging appearance of osteoma cutis into four distinct categories: single nodular, plate-like, transepidermal, and multiple miliary. The aim of this study is to describe a case of miliary osteoma cutis incidentally detected in the maxillofacial region using CBCT imaging.

## 2. Case Presentation

**Figure 1:**
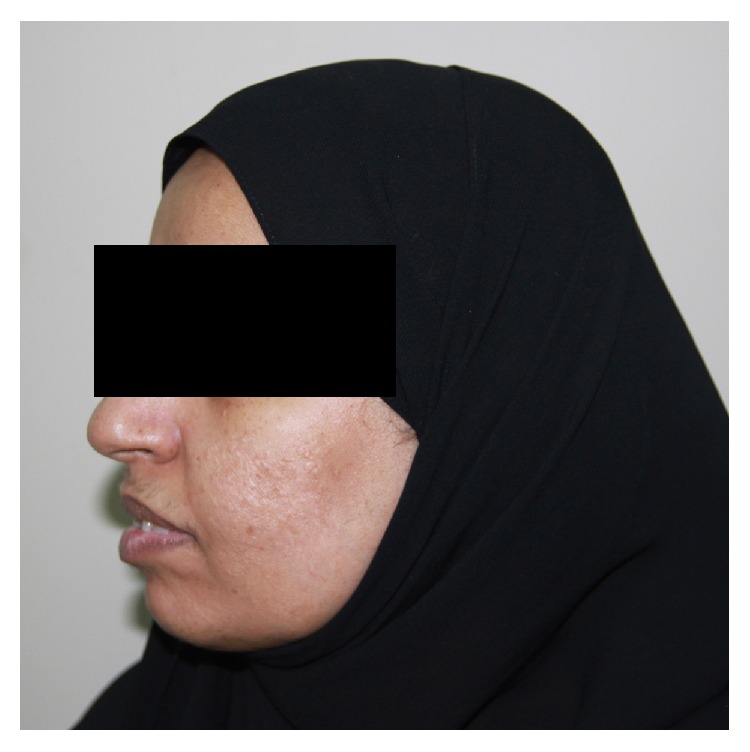
Clinical picture of the patient demonstrating the pitted skin of the cheeks.

A 45-year-old female presented to a university-based oral and maxillofacial surgery clinic complaining from pain in her jaws associated with failing fixed partial dentures and requested dental implants. Her medical history was positive for anemia and Crohn's disease. Upon examination, multiple asymptomatic papules were noted on her cheeks. The papules had hard consistency and the overlying skin appeared pitted (Figure 1). The patient was then referred to the oral and maxillofacial radiology clinic at the same university for CBCT imaging of the jaws for implant treatment planning. A Classic i-CAT® Scanner (Classic i-CAT®, Imaging Sciences International, Hatfield, PA, USA) was used to image the patient using the following parameters: 11 cm diameter × 16 cm height field-of-view (FOV), 120 peak kilovoltage (kVp), 5 milliamperes (mA), and 0.4 mm voxel size.

**Figure 2:**
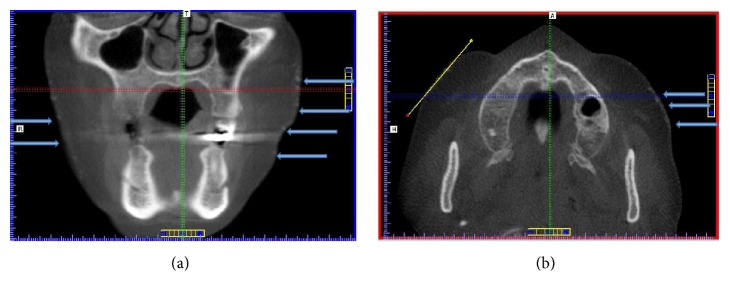
(a) CBCT coronal view. (b) CBCT axial view showing numerous radiopaque small concentric nodules in the soft tissues below the level of the zygomatic arch and lateral to the buccinator muscle.

Incidentally, multiple small round nodules of homogenous high density were noted. They appeared to be evenly dispersed within the thin layer of skin of the face (Figure 2). These imaging features are most consistent with miliary type OC, which presents as numerous lesions especially on the face of female patients. The patient was referred to the dermatology clinic for management and follow-up especially considering the patient's history of severe acne as a teenager. All laboratory blood test levels were within normal limits.

## 3. Discussion

OC is a benign condition in which soft tissue ossifications occur in the dermis layer of skin. The disease is classified into primary or secondary forms. POC is called so because it is not associated with any history of trauma or cutaneous disease and accounts for 15% of OC cases [5]. SOC, on the other hand, is associated with a known predisposing factor such as inflammation, trauma, neoplastic changes, nevi, or venous stasis [4, 11]. POC is further subdivided into two types but the subclassification is unclear and confusing. A more consensual and consistent approach to the classification of this condition is needed.

The etiology and pathogenesis of OC remain unknown and debatable. Theories range from hamartomas to nevoid tumors. Osteoblastic metaplasia of mesenchymal cells following prolonged inflammation has also been advocated [12]. Bouraoui et al., in a similar case report, suggested that acne may have resulted in scarring which in turn triggered osteoblastic metaplasia [13]. Also, Thielen et al. found an association between OC and chronic acne [14]. Moreover, approximately 85% of OC cases are believed to develop as a consequence of prolonged acne. This is reflected in the locations commonly affected by this condition, the face in females and the scalp or chest in males. OC can also occur in the breasts, extremities, and buttocks. Less frequently, OC is seen intraorally in the tongue, which is known as osteoma mucosae or osseous choristoma [15]. Some forms of OC have been reported as features of syndromes such as Albright hereditary osteodystrophy, fibrodysplasia ossificans progressiva and progressive osseous heteroplasia.

Clinically, OC presents as asymptomatic single or multiple papules, nodules, or plaques or as miliary lesions [16]. The lesions are bony hard and only occasionally cause discoloration of the skin which may become yellowish white [17]. When imaged, the lesions have a small smoothly outlined radiopaque appearance that may have a radiolucent center, with a density that is similar to bone. Their shape has been described as washer-shaped or donut- or snowflake-like and they vary in size from 0.1 cm to 5.0 cm [16].

These imaging features may mimic other calcific conditions in the facial soft tissues. Myositis ossificans, calcinosis cutis, osteoma mucosae, and dermal fillers may have the same radiographic manifestations [16, 18–20]. Subcutaneous volume enhancers such as hyaluronic acid, collagen, and silicone can also cause calcifications [21]. Surgical clips, wires, or sutures placed for procedures such as face-lifts may result in calcified nodules. Calcified phleboliths in hemangiomas may also be confused with OC [20–24]. Therefore, imaging findings should always complement adequate history taking and a thorough examination for safe arrival to the correct diagnosis.

Management of OC cases must focus on treating the underlying systemic condition if one exists. As for management of the cutaneous nodules, it is controversial and varies from no treatment to surgical excision that is usually curative without recurrence [13, 16]. Other management options include topical tretinoin, dermabrasion, needle microincision and extirpation, and resurfacing using YAG laser or CO_2_ laser [2, 16, 17, 25–27].

Volumetric imaging has revolutionized the dental practice by making it possible to visualize structures in all three dimensions. With this privilege comes the responsibility of recognizing every small detail captured within these images. It is vital to examine the entire CBCT volume for any abnormalities and to recognize and report incidental findings. This will ultimately lead to more accurate diagnosis, appropriate management, and prompt referral of patients.
